# Physical and mental health-related correlates of physical function in community dwelling older adults: a cross sectional study

**DOI:** 10.1186/1471-2318-10-6

**Published:** 2010-02-03

**Authors:** Carol Ewing Garber, Mary L Greaney, Deborah Riebe, Claudio R Nigg, Patricia A Burbank, Phillip G Clark

**Affiliations:** 1 Teachers College, Columbia University, Department of Biobehavioral Sciences, Program in Movement Sciences and Education, 525 West 120th Street, Box 199, New York, NY 10027, USA; 2 Dana Farber Cancer Institute, The Center for Community-Based Research, 44 Binney Street, Boston, MA 02115, USA; 3 The University of Rhode Island, Department of Kinesiology, 25 West Independence Way, Kingston, RI 20881, USA; 4 University of Hawaii, Department of Public Health Studies, Social and Behavioral Sciences, 1960 East West Road, Honolulu, HI 96822, USA; 5 University of Rhode Island, College of Nursing, 2 Heathman Road, White Hall, Kingston, RI 02881, USA; 6 The University of Rhode Island, Program in Gerontology, 55 Lower College Road, 100 Quinn Hall, Kingston, RI 02881, USA

## Abstract

**Background:**

Physical function is the ability to perform both basic and instrumental activities of daily living, and the ability of older adults to reside in the community depends to a large extent on their level of physical function. Multiple physical and health-related variables may differentially affect physical function, but they have not been well characterized. The purpose of this investigation was to identify and examine physical and mental health-related correlates of physical function in a sample of community-dwelling older adults.

**Methods:**

Nine hundred and four community dwelling older men (n = 263) and women (n = 641) with a mean (95% Confidence Interval) age of 76.6 (76.1, 77.1) years underwent tests of physical function (Timed Up and Go; TUG), Body Mass Index (BMI) was calculated from measured height and weight, and data were collected on self-reported health quality of life (SF-36), falls during the past 6 months, number of medications per day, depression (Geriatric Depression Scale; GDS), social support, and sociodemographic variables.

**Results:**

Subjects completed the TUG in 8.7 (8.2, 9.2) seconds and expended 6,976 (6,669, 7,284) Kcal.wk^-1 ^in physical activity. The older persons had a mean BMI of 27. 6 (27.2, 28.0), 62% took 3 or more medications per day, and14.4% had fallen one or more times over the last 6 months. Mean scores on the Mental Component Summary (MCS) was 50.6 (50.2, 51,0) and the Physical Component Summary (PCS) was 41.3 (40.8, 41.8).

Multiple sequential regression analysis showed that, after adjustment for TUG floor surface correlates of physical function included age, sex, education, physical activity (weekly energy expenditure), general health, bodily pain, number of medications taken per day, depression and Body Mass Index. Further, there is a dose response relationship such that greater degree of physical function impairment is associated with poorer scores on physical health-related variables.

**Conclusions:**

Physical function in community-dwelling older adults is associated with several physical and mental health-related factors. Further study examining the nature of the relationships between these variables is needed.

## Background

Physical function is the ability to perform both basic and instrumental activities of daily living, and the ability of older adults to reside in the community depends to a large extent on their level of physical function. As an older person experiences decline in physical function, s/he encounters increasing difficulty in engaging in the instrumental activities of daily living, and may address these difficulties by avoiding or limiting these activities. Because this decline can occur gradually, the accompanying changes in physical function may be subtle and not readily apparent to the healthcare providers, family--or even to the individual--until the person is unable to perform the activity at all.

The ability to perform a motor task (physical activity), such as those performed as part of daily living, involves the complex integration of multiple physiological systems such as the neuromotor, musculoskeletal, and the cardiorespiratory systems. The function of one or more of these systems is altered in the presence of disease or injury, and this may be clinically manifested by alterations in cognitive and motor function, physical fitness, habitual physical activity, and physical function. Each of these parameters may also be affected by a multiplicity of physical and mental health-related factors, but these parameters and their associations with physical function have not been well studied [1]. Improved understanding of variables associated with reduced physical function is important, as this may guide the development of screening tools to identify-and interventions to attenuate-declines in physical function in older persons. Thus, the purpose of this study was to identify and evaluate the correlates of physical function using a commonly utilized measure of physical function, the Timed Up and Go Test (TUG), in community dwelling older adults.

## Methods

### Subjects

Subjects of this study were community dwelling men and women 60 years of age or older from East Providence, Rhode Island and the surrounding area, and whom participated in the Study of Exercise and Nutrition in Older Rhode Islanders (SENIOR) Project [2,3]. Details of the study design, subject recruitment, evaluation, and intervention have previously been published [2,3]. Study exclusion criteria were being less than 60 years of age, living in an assisted living facility or nursing home, or the inability to give informed consent. There were no exclusions based on health status or physical function. The study was approved by and conducted in accordance with the procedures of the Institutional Review Board at The University of Rhode Island.

### Procedures

Following baseline evaluation, subjects were randomly assigned to one of four intervention groups: physical activity, diet, physical activity and diet, and a contact control condition. The intervention was delivered by print media, with periodic counselor telephone calls over a 12-month period. After the 12-month evaluation (on the completion of the intervention period), subjects received no contact or intervention for the next 12 months until the final evaluation conducted at 24 months. The data presented in this study were collected at 24 months, at the end of the 12-month no-intervention follow-up period. This time point, rather than the baseline, was selected because measurements of height and weight, depression and social support were available only at this time.

Well-trained, bilingual (English and Portuguese), older adult field interviewers collected the data in the participant's home or in the SENIOR project office. Participants answered questions concerning sociodemographics, physical and mental health, physical activity, and measurements of height, weight, and physical function were obtained.

### Sociodemographics

Sociodemographic data included sex, age, race/ethnicity, marital status, years of education, and income. Marital status was grouped as partnered (married or co-habitating) single, divorced or widow(er). Income was categorized ≤ $9,999, $10,000-$19,999, $20,000-$29,999, $30,000-$39,999, $40,00-$49,999, $50,000-$50,999, $60,000-$69,999, $70,000-$79,999, and ≥ $80,000.

### Physical Function

The Timed Up and Go Test (TUG) [4], a simple measure of physical function that involves lower extremity strength, dynamic balance, gait, and agility, was used to measure physical function. We chose a direct measurement of physical function, because self-reported measures of physical function have several limitations including that an individual must *recognize *her/his limitations, and the report biases inherent to all self-report instruments. We selected the TUG, rather than other available measures of physical function, because trained laypersons can easily administer it in the home where there may be limited space available, and it has demonstrated clinical utility in identifying physical function limitations in geriatric patients [4-6] and in a wide array of persons with diverse acute and chronic disabling conditions [7-15].

The TUG was administered using the procedures of Podsiadlo and Richardson [4]. Briefly, the subject was seated in an armless chair. The tester said, "ready, set, go" and, on the word "go", the subject was instructed to stand and walk as quickly as s/he could to a point 3 meters from the chair, turn and walk quickly back and sit down. When the buttocks were fully in contact with the chair, the test was complete and the time was recorded. Subjects completed two trials: the first was a practice trial and the completion time for the second trial was recorded. When the test was administered in the home, the test was executed on a hard floor (e.g., wood, tile) or carpeted surface if a hard floor surface was unavailable.

The classification of physical function was criterion based on the median and interquartile range of the study sample at baseline, which included 1,274 older men (n = 387) and women (n = 887) 61-97 years of age (mean age 75 ± 7 years) measured prior to randomization into study interventions. TUG scores at or below the 50^th ^percentile (≤ 8.23 seconds) represented "normal" physical function, and "physical function limitation" was considered to be present when scores were above the 75^th ^percentile (≥ 14.08 seconds). Scores between the median and the 75^th ^percentile (>8.23 to ≤ 14 seconds) were categorized as "pre-clinical physical function limitation".

This approach was chosen for several reasons: First, the existing criteria for the interpretation of the TUG were developed in mostly small, selected samples of predominantly clinical populations and limited numbers of community-dwelling older persons [4,16-18], and the applicability of these criteria to our large, diverse sample of community dwelling older adults was unknown. Second, the cut point that should be used to identify physical function limitation is unclear, ranging from ≤ 7.24 seconds [18] to ≥ 14 seconds [17] in several published studies. Illustrating the difficulty in selecting the appropriate criteria, the agreement (Tau-b) between these diverse classifications [4,16-19] ranged from 0.41 to 0.86 when applied to our sample.

Nearly all of the published criteria, including ours, classify individuals completing the TUG in less than 8.5 seconds as having normal physical function, and subjects with performance times ≥ 14 seconds would be considered to have a physical function limitation. However, there is a high degree of inconsistency in the intermediate zone between 8.5 to 14 seconds (our pre-clinical functional limitation category), with some classifying this as normal and others as abnormal. Some experts suggest that this middle range represents declining physical function and pre-clinical impairment, and identifies individuals who are on a trajectory toward disability and loss of the ability to live independently [20-22], a critical issue in community dwelling older adults, such as those in our sample. Therefore, we included a mid-range classification, which we considered to be indicative of pre-clinical impairment of physical function.

### Physical Activity

Physical activity was measured using the Yale Physical Activity Survey (YPAS), an interviewer-administered survey of habitual physical activity designed specifically for older adults [23]. Weekly energy expenditure (Kcal.week^-1^) during leisure and non-leisure physical activity was estimated as per the questionnaire scoring procedures [23]. The number of minutes per week of moderate to vigorous intensity exercise was also calculated from the YPAS. The *Physical Activity Guidelines for Americans *[24] suggest a target of 150 min.week^-1 ^of exercise for health and fitness benefit, therefore we categorized subjects engaging in ≥ 150 min.week^-1 ^of regular moderate to vigorous exercise as physically active and subjects who exercised < 150 min.week^-1 ^as physically inactive.

### Health-Related Variables

Height was measured using a portable stadiometer, and weight was measured using a calibrated portable scale; body mass index (BMI) was calculated from these measures [25]. BMI was classified as underweight (BMI <18.5 kg.m^-2^), normal weight (BMI ≥ 18.5 kg.m^-2 ^and <25 kg.m^-2^), overweight (BMI ≥ 25 kg.m^-2 ^and ≤ 29.9 kg.m^-2^), or obese (BMI > 29.9 kg.m^-2^)[25].

We measured two indirect indicators of health status: 1) the number of separate medications taken per day, and 2) the number of falls over the past 6 months. Subjects were queried about prescribed and over-the-counter medications taken regularly each day, not including "as needed" medications. Falls were assessed by self-report in response to the question, " How many times have you fallen over the past 6 months?"

The Medical Outcome Study (MOS) Short Form-36 version 2 (SF-36) was used to measure health quality of life [26,27]. The SF-36 contains eight subscales: Physical Functioning, Role-Physical, Bodily Pain, General Health, Vitality, Social Functioning, Role-Emotional, and Mental Health. Two composite scores are derived from these subscales: the Physical Component Summary and the Mental Component Summary. U.S. population-based normative scores were calculated following recommended procedures [28]. This procedure standardardizes the scores on all scales to a mean of 50, so that scores below 50 are considered below the average of the general population and scores higher than 50 above the average for the general population.

### Social Support and Depression

Social support was assessed with the Medical Outcomes Study Social Support Scale [29]. The scores were standardized and the Overall Support Index was (Social Support Scale) was calculated as the mean of each of the subscales: Emotional/Informational Support, Tangible Support, Positive Interaction, and Affection, and one additional question [29].

The Geriatric Depression Scale Short Form (GDS) was administered and scored following published procedures [30-34]. Missing scores were pro-rated by adding the product of the proportion of scores missing and the total score on the answered items to the score on the answered items [30-34]. GDS scores were interpreted as 1) no depression (GDS score ≤ 5), 2) probable depression (GDS score > 5 and ≤ 10), or 3) definite depression (GDS score >10) [30-34].

### Statistical Analyses

Statistical analyses were conducted using PASW Statistics version 17 (SPSS, Inc, Chicago, IL). Alpha levels for all analyses were set *a priori *at p < 0.05. Means and the 95% confidence intervals were calculated for continuous variables and frequencies were generated for nominal and categorical variables.

Sequential multiple regression analysis was performed in order to evaluate the associations between physical function and selected sociodemographic, physical activity, and health-related variables. Assumptions for regression were evaluated. No outliers were found using the criterion of a Mahalanobis distance of p < 0.001. No variables were found to be multicolinear upon inspection of the condition index, with a criterion for mulitcolinearity being a condition index of >15. The associations between the variables were linear as demonstrated by inspection of the bivariate scatterplots and partial regression plots. The regression analysis was performed with the continuous variable physical function (TUG score in seconds) as the dependent variable and adjusted for TUG floor surface entered in the first step of the sequential regression procedure. The remaining independent sociodemographic and health-related variables selected for the final regression model measured a unique attribute were not redundant based upon theoretical and statistical criteria, demonstrated by a significant correlation with the TUG and low correlation with other independent variables [35]. These independent variables were entered as a group into the regression equation during the second step.

A Multivariate Analysis of Covariance (MANCOVA) was performed to evaluate physical health-related variables across the three classifications of the TUG (normal, preclinical physical function limitation, and physical function limitation) in order to determine whether there was a dose-response relationship between the TUG and physical health-related variables that would be expected to co-vary with physical function. The dependent variables included in the MANOVA were General Health Subscale, BMI, Physical Component Summary Score, Physical Function Subscale, Role-Physical Subscale, and Weekly Energy Expenditure. Covariates included age, sex, race/ethnicity, education, intervention assignment, and TUG floor surface. Univariate One-Way Analyses of Covariance and pairwise comparisons with Bonferroni adjustment for multiple comparisons were also conducted where there were significant multivariate effects.

## Results

Nine hundred and four older adults (263 men, 641 women) completed the TUG at 24 months. The demographic characteristics of these individuals are presented in Table 1. Almost 14% did not complete high school, and 22.4% were from under-represented racial and ethnic groups. Of the 478 subjects reporting income, 55% had an annual household income less of than $20,000, and 13.4% had an income of $40,000 or more per year.

**Table 1:** Sociodemographic Characteristics of 903 Older Adults

Variable	Mean (95% Confidence Interval) or Number of Subjects
Age (years)	76.6 (76.1, 77.1)
Education (years)	13.0 (12.7,13.2)
Ethnicity/Race (n)	
White	692
Black	20
Portuguese/Cape Verdean	124
Other	47
Marital Status (n)	
Married/Partnered	406
Widow(er)	355
Divorced/Separated	105
Single	35

The results of the evaluations of physical activity and health-related variables are shown in Table 2. The older persons ranged from underweight to obese: 0.5% of subjects were underweight, 31.6% were of normal weight, 39.2% were overweight, and 28.7% were obese. Subjects reported a wide range of leisure and non-leisure physical activity, but only 2.5% met current recommendations for exercise[24]. Table 3 shows the categorization of physical function of our subjects.

**Table 2:** Health and Physical Activity Status of 904 Older Adults at 24 Months

Variable	Mean (95% Confidence Interval)
TUG (seconds)	8.7 (8.2, 9.2)
Physical Activity	
Weekly Energy Expenditure	
Kcal.wk^-1^	6,976 (6,669, 7.284)
Exercise Time (min.wk^-1^)	238.3 (227, 250)
BMI (kg.m^-2^)	27.6 (27.2, 28.0)
Medications (number per day)	3.8 (3.5, 4.0)
Falls in the past 6 months (number)	0.22 (0.17, 0.26)
Geriatric Depression Score	1.3 (1.1,1.5)
Social Support Scale	84.3 (82.7, 86.0)
SF-36 Scores	
Mental Component Summary	50.6 (50.2, 51.0)
Physical Component Summary	41.3 (40.8, 41.8)
General Health	53.4 (52.8, 54.0)
Bodily Pain	40.0 (39.8, 40.2)
Mental Health	54.3 (53.7, 54.9)
Physical Function	43.8 (43.1, 44.6)
Role Emotional	38.3 (37.9, 38.5)
Role Physical	37.7 (37.3, 38.1)
Social Functioning	53.6 (52.0, 53.2)
Vitality	45.3 (45.0, 45.5)

**Table 3:** Classification of Physical Function Limitation Measured by the Timed Up and Go Test

Criterion	Number (Percent)
Normal physical function (≤ 8.23 Seconds)	519 (57.4%)
Pre-clinical physical function limitation (8.23-14 seconds)	199 (22.0%)
Physical function limitation (≥ 14 Seconds)	186 (20.6%)

The mean scores on Physical Component Score (PCS), Physical Function (PFS), Role Physical (RPS), Role Emotional (RES), Bodily Pain (BPS), and Vitality (VS) were significantly lower than the norm, and the Mental Component Score (MCS), General Health (GHS), Mental Health (MHS), Social Functioning (SFS) Scores were significantly above the norm for the general population. Subjects had good social support, with the standardized scores substantially above the median.

The results of the linear regression analysis are shown in Table 4. After adjustment for TUG floor surface, the independent predictors of physical function were general health, age (years), sex, bodily pain, education (years), BMI, medications (number per day), GDS Score, and total weekly energy expenditure (Kcal.wk^-1^), with each variable significantly (p ≤ 0.001) contributing to the increase in R^2^. With the addition of each of these variables into the equation, R was significantly different from zero. Additional sociodemographic and health-related variables did not reliably improve R^2^, and therefore, were excluded from the final regression model.

**Table 4:** Sequential Regression Of Sociodemographic, Health-Related, And Physical Activity Variables On Physical Function In Older Adults

Variable			Coefficients			
			B	SEE	β	p ≤
Constant			-7.771	3.895		.046
Sex			.816	.396	.063	.039
Age (years)			.196	.030	.214	.000
BMI (kg.m^-2^)			.163	.036	.146	.000
Education (years)			-.151	.062	-.077	.014
Bodily Pain			.103	.042	.076	.014
Energy Expenditure (Kcal.wk^-1 ^)			.000	.000	-.162	.000
Medications (number per day)			.223	.060	.124	.000
GDS Score			.297	.103	.106	.004
General Health			-.064	.027	-.090	.020
Floor Surface			-.865	.253	-.105	.001
Model Summary						
			**ANOVA**			
**R**	**R^2^**	**Adjusted R^2^**	**SEE**	**F Change**	**df**	**P ≤**

.503^b^	.253	.244	5.07	27.8	10, 819	0.0001

Dependent variable is TUG (seconds)

The MANCOVA showed significant multivariate effects of TUG classification (Pillai's Trace = 0.328; F = 24.596, p ≤ 0.0001), and significant (p ≤ 0.0001) between subject effects for General Health Subscale, BMI, PCS, Physical Function Subscale, Role-Physical Subscale, and Weekly Energy Expenditure. Table 5 shows the results of the univariate and bivariate analyses across the classifications of the TUG (normal, pre-clinical physical function limitation, physical function limitation) for each of the physical health-related variables. There was a significant, dose-response relationship for each variable, such that the scores on the physical health-related variables worsened as the degree of impairment by the TUG increased.

**Table 5:** Adjusted Physical Health-Related Variables Across Categories of Physical Function Limitation

	Physical Function Limitation			Univariate Comparisons		
**Variable**	**Normal**	**Pre-clinical limitation**	**Limitation**	**F**	**df**	**p ≤**
General Health	55.9	52.4*	49.7*^#^	50.87	2, 783	0.0001
	(55.2, 56.4)	(51.1, 54.0)	(48.4, 51.0)			
BMI (kg.m^-2^)	26.9	28.4*	29.9*^#^	21.0	2, 783	0.0001
	(26.4, 27.4)	(27.7, 29.1)	(29.1, 30.7)			
Physical	56.7	52.3*	48.6*^#^	72.87	2, 783	0.0001
Component Summary	(56.2, 57.3)	(51.1, 53.5)	(47.2, 49.9)			
Physical Function	49.6	42.7*	34.0*^#^	158.94	2, 783	0.0001
	(48.9, 50.3)	(41.3, 44.0)	(32.4, 35.6)			
Role-Physical	39.9	37.1*	33.6*^#^	93.56	2, 783	0.0001
	(39.5, 40.2)	(36.3, 38.0)	(32.4, 35.6)			
Weekly Energy Expenditure (Kcal.wk^-1^)	7,851	6,363*	4,314*^#^	38.57	2, 783	0.0001
	(7,523, 8,179)	(5,828, 6898)	(3,881, 4,750)			

Values are means and (95% confidence interval)

Adjusted for age, sex, race/ethnicity, education, floor surface, and intervention assignment

* Significantly different (p < 0.01) from normal

# Significantly different (p < 0.01) from preclinical physical function limitation

## Discussion

This study examined sociodemographic and health-related variables to learn more about the associations between these variables, and to identify possible influences on measured physical function in community-dwelling older adults. These results from the SENIOR study demonstrate that there are multiple factors associated with physical function, which encompass sociodemographics, physical and mental health. These findings complement the results of the U.S. Longitudinal Study of Aging [36] that reported physical and mental health were predictive of declining physical function and mortality.

### Sociodemographic Factors

The results of the regression analysis showed that age, sex, and education level are associated with physical function, with poorer TUG performance times with increasing age, and in women and older persons with lower levels of education. Others [16,19,37,38] have also demonstrated that increasing age is associated with poorer TUG performance time. Sampson and colleagues found that the poorer TUG scores in older persons was associated with poorer muscular strength and power [39], suggesting the TUG mirrors the physiological changes that occur with aging and physical inactivity.

Population studies report more mobility limitations in older women compared with than older men [40,41]. Few studies have examined sex differences in performance times on the TUG, but this is likely due to the small numbers of men in the study samples. Vereeck and colleagues [42] found that older women have longer TUG times and poorer standing balance compared with older men. The Tromso study [43] found there was a significant difference between the mean TUG time in older men who fell compared with older women who fell. Interestingly, a recent report from the Rancho Bernardo Study [44], a longitudinal study of community dwelling men and women, reported no differences in TUG performance time between men and women, but did find that low 25-hydroxyvitamin D levels were associated with poorer performance on the TUG and Timed Chair Stand Test in women, but not men, suggesting that the factors affecting physical function may differ by sex.

Level of education is a surrogate measure of socioeconomic position, and our study shows that lower educational attainment is associated with poorer performance on the TUG. These results are supported by large population studies conducted in the United States [45], Sweden [41], Denmark [46], and the United Kingdom [47] that report greater mobility limitations in persons of lower socioeconomic position, although none of these studies have used the TUG to measure physical function.

### Physical Health

Several measures representing different aspects of physical health were independent correlates of physical function. These included general health, bodily pain, number of medications taken per day, physical activity (weekly energy expenditure), and body mass index. The association between self perceived health and physical function is supported by other studies, although these studies did not measure physical function with the TUG [6,22,48] In the SENIOR subjects, taking more medications was also associated with poorer physical function. It is not surprising that the number of medications per day are associated with physical function, because medication use is indicant of such things as health status, medical care, polypharmacy, and adverse outcomes (e.g., falls) [49].

Higher BMI was also associated with poorer physical function, confirming other studies of older persons where elevated BMI was associated with self-reported limitations in physical function [40,50,51]. This may be because overweight can affect the desire and motivation to engage in physical activity and physical activity behavior [52,53]. Other studies have shown that BMI that is low (< 18.5) and high (>25) are associated with poorer health [52]. Our results did not show this bifurcated relationship between BMI and physical function, but this may be due to the limited number of persons with low BMI in our sample.

The weekly energy expended by leisure and non-leisure physical activity was an independent correlate of physical function. These results confirm the findings of previous studies that have shown individuals who are more physically active have better physical function and are less likely to have physical function impairment or disability [52,54].

Consistent with other studies, bodily pain was also associated with TUG performance times. A population study in Western Australia [38] found that the TUG, PCS and MCS were all lower in older persons with self-reported pain in the lower extremities. Other studies using the TUG or other measures of physical function have also reported this association with pain [55-58].

### Mental Health

Higher scores on the Geriatric Depression Scale were associated with poorer physical function in our sample of community-dwelling older adults. While the GDS is intended to screen for depression and does not indicate a clinical diagnosis of depression, it is nonetheless interesting to note that apparent poorer mental health co-exists with greater degrees of physical impairment, as indicated by poorer performance on the TUG. Several studies have shown that depressed persons have increased mortality and morbidity [59-61], and are more likely to be physically inactive [52,62]. Impairment in activities of daily living, slower walking speeds, slower walking speeds, poorer self rated health, poor cognitive status, and two or more clinic visits in the past month were identified as risk factors for depression and falling in older primary care clinic patients [63]. Tinetti and colleagues showed that depression or anxiety, reduced physical function (repeated chair stands), and decreased upper extremity strength were associated with a higher risk of urinary incontinence, falling, and functional dependence. On the other hand, a longitudinal study of pre-clinical disability conducted in community dwelling older persons [21] showed that depression, living alone, the number of chronic diseases were not associated with preclinical disability, while difficulty in walking and climbing stairs, task modification for stair climbing were significant predictors.

### Classification of Physical Function

We used a trichotomous classification of physical function developed from our baseline data. The results of the MANOVA analyses showed significant trends across the three categories of physical function for several physical health-related variables, including general health, BMI, PCS, physical function, role-physical, and physical activity, supporting the validity of these categories. The magnitude of difference between the categories for each of these physical health-related variables is comparatively small, and it is unknown if these differences are clinically important. However, the consistency of the differences across these several variables supports the existence of a real, albeit very modest, difference. Nonetheless, the inclusion of an intermediate classification may be useful in identifying persons who have pre-clinical physical function impairment. This is important, because there is increasing evidence for a period termed "preclinical disability" where individuals experience some physical function limitation, but are not disabled [20-22,64,65]. During this "preclinical" phase of the downward trajectory in the disablement process, persons may modify physical tasks such as slowing walking speed, resting while climbing stairs, or perform activities while sitting rather than standing, and/or they may restrict or avoid activities where they experience some difficulty [66]. Often, because of these compensatory actions, the physical function limitations may not be readily apparent. However, being able to identify these persons who are at high risk of becoming disabled and who may be responsive to interventions could be critical in preventing or delaying disability [21,22]. Self report methods have validity in identifying "preclinical disability" and future disability, and some tests of physical function have been able to predict future disability in longitudinal studies [20-22,64], but there are few clinical criteria by which to identify preclinical physical function limitations using physical performance tests, such as the TUG. While additional study is needed to determine whether our middle category of TUG performance is useful in this regard, the results of this study suggest the possibility of usefulness.

Nearly all of the existing classification schemes for the TUG are dichotomous, that is, they identify a cut point for "abnormal and normal" physical function, but creating much confusion to the interpretation of the TUG, these cut points vary considerably [16-19,37]. Nearly all of the classification criteria, including ours, identifies a TUG score of ≤ 8.5 seconds as normal and a score of ≥ 14 seconds as abnormal. It is in the intermediate range of > 8.5 seconds and <14 seconds where there is disagreement, and it is notable that this range of uncertainly falls within our middle category (8.3-14 seconds), lending additional credibility to including this additional classification category.

### Study Limitations

The data presented here are a secondary analysis of data collected as part of a randomized intervention study (SENIOR Study). As explained previously in the methods, the data were collected 12 months following the intervention was completed. Although there were some persistent treatment effects at this time point [67,68], there is still a wide range in the variables of interest, and, we believe, sufficient to answer the research questions posed in this manuscript.

The lack of standardization of the chair and floor surface used to administer the TUG was a notable limitation of this study. Other studies have shown that chair characteristics can affect the TUG score [69] and, in this study, floor surface was a significant covariate of TUG scores. These factors undoubtedly contributed to the substantial variability in TUG scores, and most likely attenuated the magnitude of the associations observed. Inter-rater reliability of the TUG has been reported to be excellent in other studies (r ≥ 0.92) [17,70-73], although inexperience of the raters was associated with lower inter-rater reliability (r = 0.87) [7]. Our survey team was well trained, which should have minimized the degree of variability introduced due to inter-rater factors.

Sampling and adherence factors may have contributed the presence of mild to moderate selection biases in the study, with the result of an under-estimation of the degree of associations between some variables and physical function. The study sample was a voluntary one and may not be representative of the population of community-dwelling older adults with respect to the variables measured. For example, it is possible that less healthy and more disabled elders did not volunteer for the study, in spite of the lack of exclusion criteria based on health and functional status. Extensive efforts to recruit from all segments of the community-dwelling older adult population in order to minimize sampling biases in our sample, but efforts were not entirely successful in obtaining a fully representative sample [74]. The SENIOR Project had a 24.2% drop out rate at 24 months, with the subjects who dropped out tending to be men, more sedentary, less educated, and with poorer self-reported health [67,74]. Thus, our sample was somewhat healthier and more active than the general population.

## Conclusions

The SENIOR study shows that physical function covaries with a range of multiple physical and mental health-related variables, and there is a dose response relationship across levels of physical function for physical health-related variables. These observed relationships are important because they identify areas for future, more complex study of the nature of these relationships including potential mediator and moderator relationships and causal pathways between these variables. Further, it is interesting to note that poor physical function and each of the health-related variables associated with physical function are predictors of increased morbidity and mortality in older persons [24,75-78], and further study is needed to evaluate the nature of the relationships between these variables and morbidity and mortality outcomes. The correlates of physical function identified in the SENIOR study include both modifiable and un-modifiable factors, suggesting that physical function can be improved from interventions addressing these factors.

In conclusion, physical function in community dwelling older adults is associated with several physical and mental health-related factors. Further work evaluating the nature of the associations between these variables and physical function and how changes in these variables may co-vary is needed.

## Competing interests

The authors declare that they have no competing interests.

## Authors' contributions

CEG conceived of the study, conducted the statistical analysis and led the writing of the manuscript. MLG contributed to the conception of the study, the data collection, and writing of the manuscript. DR contributed to the conception of the study, the data collection and writing of the manuscript. CRN contributed to the data collection and manuscript writing. PAB contributed to the study design and data collection. PGC is the SENIOR study principal investigator and contributed to writing of the manuscript. All authors read and approved the final manuscript.

## Pre-publication history

The pre-publication history for this paper can be accessed here:

http://www.biomedcentral.com/1471-2318/10/6/prepub
